# High Intensity Resistance Training Methods with and without Protein Supplementation to Fight Cardiometabolic Risk in Middle-Aged Males: A Randomized Controlled Trial

**DOI:** 10.1155/2016/9705287

**Published:** 2016-01-18

**Authors:** Wolfgang Kemmler, Andreas Wittke, Michael Bebenek, Michael Fröhlich, Simon von Stengel

**Affiliations:** ^1^Institute of Medical Physics, University of Erlangen-Nürnberg, Henkestrasse 91, 91052 Erlangen, Germany; ^2^Department of Sports Science, University of Kaiserslautern, Erwin-Schrödinger-Straße, 67663 Kaiserslautern, Germany

## Abstract

Time-effective protocols may potentially increase people's compliance with exercise. The purpose of this paper was to compare the relative effects of 16 weeks of high intensity (resistance) training (HIT) with and without protein supplementation (HIT&P) and HVHIT (high volume/high intensity training) versus a nontraining control group on cardiometabolic risk factors. One hundred and twenty untrained males 30–50 years old were randomly assigned to 3 subgroups: (a) a HIT group; (b) a HIT&P group, and (c) a waiting-control group (phase I) that crossed over to (d) high volume/high intensity training (HVHIT) during the second study phase. HIT was defined as “single set to failure protocol” while HVHIT consistently applied two sets. Protein supplementation provided an overall intake of 1.5 g/kg/body mass. Primary study endpoint was the metabolic syndrome *Z*-Score (MetS-*Z*-Score). MetS-*Z*-Score significantly improved in all exercise groups (*p* ≤ 0.001) with no significant difference between HIT, HIT&P, and HVHIT (*p* ≥ 0.829). However, all the exercise groups differed significantly from the CG (*p* < 0.001) which deteriorated significantly (*p* = 0.039). In conclusion, all exercise protocols were similarly effective in improving cardiometabolic risk factors. Thus, HIT may be the best choice for people with low time budgets looking to improve their cardiometabolic health.

## 1. Introduction

Most employed persons stated time constraints as the main obstacle to frequent exercise training; thus, time-effective exercise protocols may potentially increase people's compliance with exercise or training interventions. With respect to resistance exercise, low volume, high intensity training (HIT) protocols seem to be the most time-efficient way to improve muscle mass and strength. Although the general relevance of resistance exercise in cardiometabolic prevention is undisputed [1–4], the relative effect of HIT compared with more time consuming high volume training (HVHIT) protocols on dedicated cardiometabolic indices and risk factors is very scarce. Revisiting the ongoing debate, resistance exercise with higher volume seems to be more effective on muscular parameters [5–9] compared with HIT. Additional protein supplementation may relevantly increase the hypertrophic effect of HIT [10] and may therefore eliminate LBM-induced differences [11] of cardiometabolic parameters. Thus, the purpose of this paper was to compare the relative effects of HIT, HIT, and protein supplementation (HIT&P) and HVHIT (high volume/high intensity training) versus a nontraining control group on cardiometabolic risk factors in untrained middle-aged men. Our primary hypothesis was that all exercise programs resulted in significantly more favorable changes of the metabolic syndrome compared with the nontraining control group. Our secondary hypotheses were that (a) the HVHIT and (b) the HIT&P protocols are significantly more effective than the HIT protocol.

## 2. Materials and Methods

### 2.1. Experimental Design

The physical adaptions in Untrained on Strength and Heart (PUSH) study is a 22-week randomized controlled exercise trial with untrained middle-aged males. The study compared the effects of different resistance exercise protocols with and without protein supplementation versus sedentary controls on muscle mass, strength, and cardiometabolic parameters. In this paper, we focus on the latter. The study was initiated by the Institute of Medical Physics (IMP), Friedrich Alexander-University Erlangen-Nürnberg (FAU), Germany. The study complied with the Declaration of Helsinki “Ethical Principles for Medical Research Involving Human Subjects” and was approved by the ethics committee of the FAU (Ethikantrag number 53_12 B) and the Federal Bureau of Radiation Protection (Z5-22462/2-2012-060). After detailed information, all participants gave written informed consent. The study was registered under ClinicalTrials.gov (NCT01766791).

### 2.2. Participants

**Figure 1:**
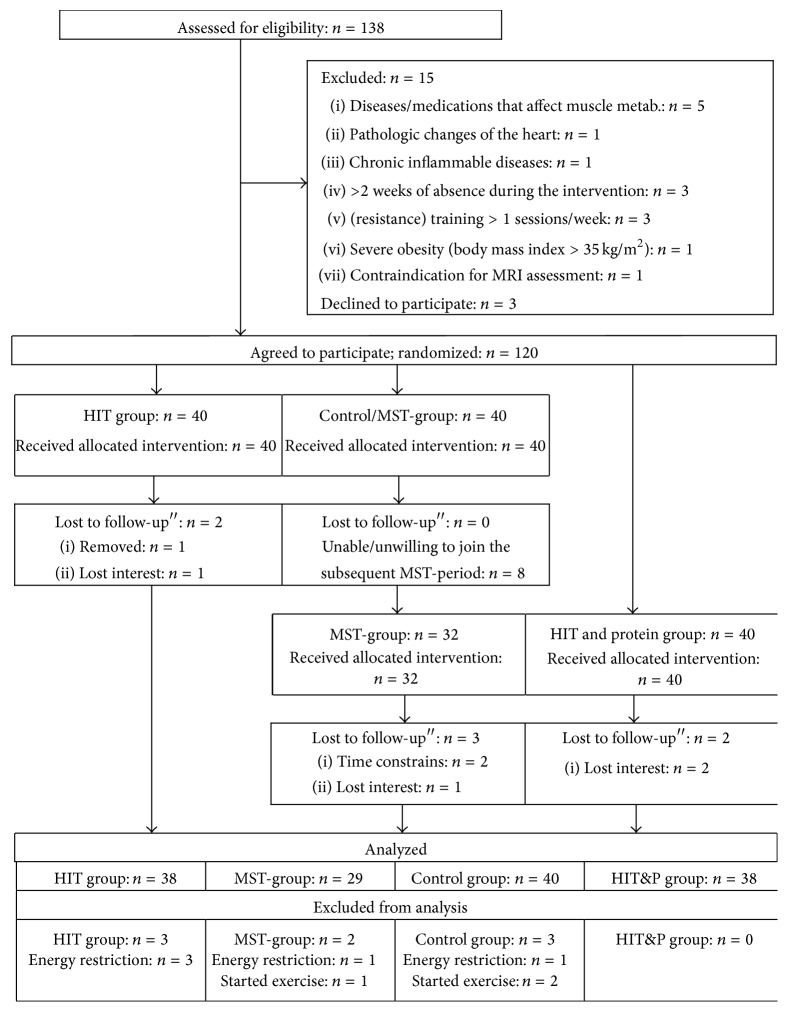
Flow chart of the study of the PUSH study. Of importance, the inactive control group of the first study period “crossed over” and performed the HVT during study period II.

**Figure 2:**
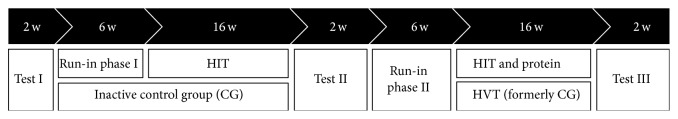
Study design of the PUSH study. It is pointed out for a better understanding that the inactive control group of the first study period performed the HVT during study period II (cross-over).

Figure 1 shows the participant flow of the study. Using the citizens register, 2,000 randomly selected men between 30 and 50 years living in the area of Erlangen, Germany, were contacted between October and December 2012. Personalized letters gave detailed study information including the key eligibility criteria for the study. A hundred thirty-eight men responded and were assessed for eligibility. Of these, 15 subjects had to be excluded due to the criteria of (a) “being untrained” (i.e., ≤1 resistance exercise session/week; ≤2 total exercise sessions/week during the last 2 years; *n* = 3), (b) inflammatory diseases and pathological changes of the heart (*n* = 2), (c) diseases affecting the cardiovascular system and muscle or medication affecting muscle metabolism (*n* = 5), (d) severe obesity (BMI > 35 kg/m^2^; *n* = 1), (e) >2 weeks of absence during the interventional period (*n* = 3), and (f) contraindication for MRI-assessment (*n* = 1). Of the remaining 123 males, three subjects were unwilling to join the randomization procedure and quit the study before randomization. Thus, 120 subjects were randomly assigned (block randomization stratified for age) to 3 subgroups: (a) high intensity (resistance exercise) training (HIT) group, (b) HIT and protein supplementation, (c) nontraining waiting-control group (period I), and (d) high-volume (resistance exercise) training (HVHIT) (Figures 1 and 2).

### 2.3. Primary Study Endpoints

(i) Metabolic syndrome (MetS) *Z*-Score according to Johnson et al. [12].

Secondary study endpoints were as follows:

- Criteria constituting or related to the NCEP ATP III METS criteria [13]:
  - Waist circumference.
  - Mean arterial pressure (MAP).
  - Fasting glucose.
  - Triglycerides.
- Total cholesterol/HDL-cholesterol-rate.
- Abdominal body fat (%).

Confounding/explanatory study endpoint was

- lean body mass (LBM).

### 2.4. Assessments

All assessments were performed in a blinded fashion. Staff were not informed and were not allowed to ask about the group status. Participants were consistently tested by the same researcher at the same time of day (±1 h) at baseline and follow-up.

Height (Holtain, Crymych Dyfed., Great Britain) and body mass were measured using calibrated devices (InBody 230, Seoul, Korea). Waist circumference was measured as the minimum circumference between the distal end of the rib cage and the top of the iliac crest along the midaxillary line. Fat and fat free mass (LBM) were assessed by Dual Energy X-Ray Absorptiometry (DXA, QDR 4500a, Discovery-upgrade; Hologic Inc., Bedford, USA) using standard protocols [14]. LBM was defined as fat-free mass (including bone) of the whole body. Abdominal fat was specifically segmented between the lower edge of the 12th rib and the upper edge of the iliac crest.

Blood was sampled in the morning (7:00 to 9:00) after an overnight fast in a sitting position from an antecubital vein. Serum samples were centrifuged at 3000 RPM for 20 minutes and immediately analyzed by the Medical Department of the FAU. Glucose, total cholesterol, HDL- and LDL-cholesterol, and triglycerides (Olympus Diagnostica GmbH, Hamburg, Germany) were determined.

Blood pressure (RR) was determined immediately after the ≈5 min DXA scan remaining in a resting, lying position with an automatic oscillometric device (Bosco, Bosch, Jungingen, Germany). Subjects were requested to arrive in a relaxed, nonfasting condition but having refrained from coffee or tea for at least 2 hours prior to testing. Mean arterial pressure (MAP) was calculated (diastolic RR + diastolic RR + systolic RR)/3.

In the present paper, we followed the NCEP-ATP III Criteria [15] of the metabolic syndrome and used the MetS-*Z*-Score calculation proposed by Johnson et al. [12]. As per this approach [12], the ATP-III cut-off point for a male population and the corresponding baseline standard deviation (SD) of the entire PUSH-cohort were applied for each MetS parameter of the individual data. In detail, the *Z*-Score was calculated using [(40 − HDL-cholesterol)/SD HDL-C] + [(triglycerides − 150)/SD TriGly] + [(Glucose − 100)/SD Glucose] + [(waist circumference − 88)/SD WC] + [(MAP − 100)/SD MAP]. Negative *Z*-Scores and negative *Z*-Score changes can be considered as favorable.

Baseline characteristics and confounding factors (i.e., lifestyle, diseases, medication, and physical activity) were assessed at baseline and follow-up by standardized questionnaires and personal interviews.

The participants' dietary intake was assessed pre- and posttrial by a 4-day dietary protocol maintained by all the participants. The consumed food was analyzed using the Freiburger Ernährungs-Protokoll (Freiburger Nutrition Protocol) (Nutri-Science, Hausach, Germany).

The sample size calculation of the PUSH study was based on the study parameter “fat-free, cross-sectional area (i.e., muscle density) of the midthigh,” which is not covered in this paper. However, with respect to the present study endpoint, the generated sample size (at least *n* = 29 per group, Figure 1) allowed us to detect a MetS-*Z*-Score between group difference of 1 ± 1.25 score points with a power (1 − *β*) of 0.86 (*α* = 0.05).

All the subjects who took part in the follow-up measurements were included in the analysis independently of their compliance. However, due to severe infringements of the study protocol we excluded subjects who (a) decreased energy intake > 10% during the interventional period or/and (b) started relevant endurance (i.e., running ≥20 km/week) or resistance exercise training (≥1 session/week) outside the PUSH study.

### 2.5. Intervention

#### 2.5.1. Exercise Program

Figure 2 shows the study design. The first study period started in early (HIT) or late (CG) January 2013 and focused on the HIT and the control group. After 2 weeks of introduction and briefing and 4 weeks of conditioning applying 1-2 sessions/week, 10 exercises with consistent 2 sets of 10–15 repetitions (reps), and nonfailure (maximum effort minus 2-3 reps) the HIT groups started their 16 week single-set-to-failure protocol as described below. Meanwhile the control group was requested to maintain their lifestyle and physical activity. Follow-up tests for the HIT group were conducted during early July, while the corresponding tests for the CG were conducted in late July. After a holiday break during August, the CG group started their HVHIT phase in early September with no further baseline tests, while the HIT and protein groups conducted their initial assessment during the 2nd and 3rd weeks of September. Following the procedure of the HIT group, both exercise groups performed an identical 6-week conditioning program followed by 16 weeks of HIT or HVHIT exercise, as described below. Finally, both groups underwent follow-up assessments in early (HVT) or late (HIT&P) February.

The exercise program generally consisted of two to (rarely) three consistently supervised sessions/week. All the main muscle groups were addressed by 10–13 exercises/session taken from a pool of 17 exercises (latissimus back and front pulleys, front chin ups, seated rowing, back extension, inverse fly, hyperextension, sitting bench press, shoulder-press, military press, butterfly with extended arms, crunches, leg press, leg extension, leg curls, leg adduction, and abduction) conducted on resistance devices (MedX, Ocala, FL, USA). Intensity of the exercise was prescribed as a range of repetitions (e.g., 6–8 reps) that had to be accomplished under the premise of work to momentary muscular failure (MMF). In doing so, subjects were asked to generally focus on the lower range of this prescription. “Warm-up” of the muscle group was conducted at the corresponding resistance machine with low intensity (50%) and few reps (6–8). This procedure was performed only once per muscle group, however; thus, one warm-up set was performed for each synergistic block.

HIT was defined as “single set to failure protocol”. Single set, however, refers to “exercises” not to “muscle groups”; thus, the same muscle group may be addressed by several exercises that were performed once. HVHIT applied the identical exercise protocol but with each exercise always being performed twice in order to properly evaluate the isolated effect of higher volume per session. Of importance, the HVHIT was organized as a circuit; that is, the second set/exercise was not conducted immediately after the first set but after the first bout of all exercises.

After the 6-week conditioning period, a linearly periodized resistance exercise program with Four 4-week phases with each 4th week as a rest week was implemented for a further 16 weeks. The number of repetitions was steadily decreased from 8–10 to 3–5 reps over all the phases.

Phase 2 focused on work to momentary muscular failure (MMF) with rest periods of 2-3 min between exercises/sets. However, after week 8 we reduced the rest periods to 1 min between exercises in order to further increase general exhaustion under the protocol. Time under tension (TUT) was consistently prescribed at 2 s (concentric)-1 sec (isometric)-2 s (eccentric).

During phase 3, we added a superset strategy with one session/week prescribing a synergistic approach (4 blocks of 2–4 different exercises for the same muscle group consecutively) while the other sessions/week focused on an antagonistic approach (5 blocks of one exercise each for agonist and antagonist consecutively). Rest periods between the synergistic or antagonistic exercises were ≤1 min while rest periods between the blocks were 2 min. Movement velocity varied from (TUT) “explosive” – 1 s-2 s for the higher repetition ranges (8–10 reps) to 3 s–1 s–3 s for the lower repetition ranges (3-4 reps).

During phase 4, we also enhanced the muscle effort by prescribing further reps with reduced load (−10–15%) immediately after the initial workout to MMF (“drop sets)”. During phase 5, each second session, load was reduced twice (e.g., −10% work to MMF and again −10% work to MMF). Rest periods between all exercises either within or between the blocks were 2 min for the first 4 weeks of this period and ≤1 min within the synergistic/antagonistic blocks and 90 sec–2 min between the blocks for the second 4-week period of this phase. Movement velocity during phases 4 and 5 was consistently prescribed at (TUT) 2 s-1 s-2 s, with the subjects being required to work with a 3-1-3 TUT for the forced repetition with reduced load.

Training logs were provided for all the training phases. Besides proper completion of the logs, participants were asked to list the net exercise time and their rate of perceived exertion (Borg CR 10 Scale) [16] for the corresponding session. Further training attendance was precisely determined by chip cards that gave subjects entrance to the gym.

#### 2.5.2. Supplementation

Based on dietary protocols, 32 participants of the HIT&P group demonstrated daily protein intakes of <1.5 g/kg bodyweight. These participants were provided with protein powder (protein4you, Saarlouis, Germany) in order to realize an individual protein supply of at least 1.5 g/kg body mass per day. This supplement consisted of multicomponent protein (whey, casein, egg, and soya) with a chemical score of 156. Hundred (100) g contained 76.5 g of protein, 3.9 g of carbohydrates, and 3.2 g of fat resulting in a calorific value of 363 kcal/100 g protein powder. One portion of 30 g, for example, included 3 g of L-Leucine and was enriched with 500 mg L-Carnitine. Subjects were requested to ingest supplements after exercise and, if applicable, to split doses when protein supplement intake increased to 30 g/d. Compliance with prescribed protein powder intake was regularly requested during the HIT sessions of this exercise group.

### 2.6. Statistical Analysis

Baseline characteristics were reported as means (MV) with standard deviations (SD). Normal distribution of variables was checked graphically and statistically (Shapiro-Wilks-Test). For normally distributed variables, differences within groups were analyzed with paired *t*-tests; otherwise, the Wilcoxon sign-test was used. In parallel, significance for group differences of normally distributed variables was checked with ANOVA; otherwise, the Wald-Wolfowitz-Test was used. Tests were consistently adjusted to corresponding baseline values. Corresponding pairwise tests were conducted with the “Scheffé” test procedure. In order to check interactions between dependent and confounding parameters, linear regression models were used. All the tests were 2-sided using a significance level of 0.05. Multiple testing corrections for the secondary study endpoints were not applied. SPSS 23.0 (SPSS Inc., Chicago, IL) was used for all statistical procedures.

## 3. Results

Table 1 gives the pretest characteristics of the participants. No relevant differences (*p* > 0.30) were observed with respect to anthropometric parameters or factors (e.g., lifestyle including physical activity, medication, and diseases) that may have affected the study results.

In summary seven subjects (HIT: *n* = 2; HIT&P = 2, HVHIT = 3, CG: *n* = 0) were lost to follow-up, eight subjects dropped out after their control phase and did not join the HVHIT period. The reasons given for withdrawal were (a) relocation (*n* = 2), (b) time constraints due to paternity or occupational changes (*n* = 3), (c) loss of interest (*n* = 1), and (d) starting resistance exercise during their control phase (*n* = 2). Application of the study protocol led to 8 subjects being excluded (Figure 1) due to an energy restriction of >10% (*n* = 5), start of endurance (HVHIT: *n* = 1), or resistance exercise training (CG: *n* = 2). Apart from these subjects, no further participant of the CG reported changes of physical activity during the study period (I).

Actual average protein supply (dietary intake and protein supplements) of the HIT&P group ensured the intended intake and averaged 1.62 ± 0.12 g/d kg bodyweight (1.44–1.86 g/d).

Attendance rates for all exercise groups were high (HIT: 94 ± 6%; HIT&P: 96 ± 4%; HVHIT: 95 ± 6%) and comparable (*p* = 0.078) between the groups. Rate of perceived exertion (RPE) per session (recreational weeks and 6-week conditioning phase excluded) was also similar (*p* = 0.886) between the groups (6.7 ± 1.1 to 6.8 ± 1.0 on Borg CR 10; 7 = very hard). Average net duration of the exercise training per se varied significantly between HIT and HIT&P (36.6 ± 2.4 min) versus HVHIT (74.7 ± 2.1 min). No injuries occurred during the exercise sessions. Furthermore, no changes of diseases or medication were reported during the intervention period.

### 3.1. Primary Study Endpoints

**Figure 3:**
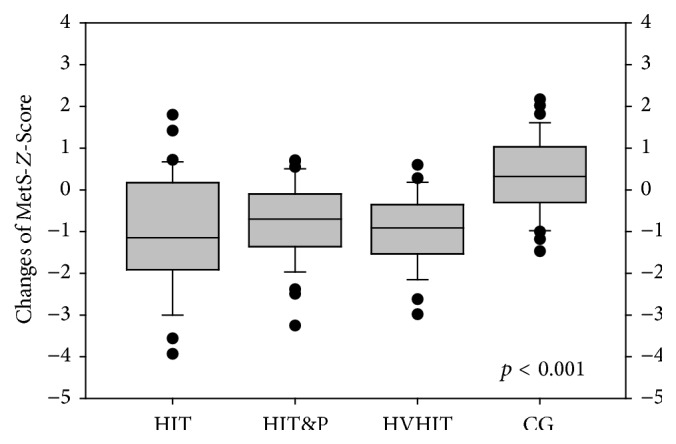
MetS-*Z*-Score changes.

At baseline, there were no significant differences for METS-*Z*-Score between the groups (Table 2). After 22 weeks of resistance exercise, the MetS-*Z*-Score improved significantly in all exercise groups (*p* ≤ 0.001) with no significant difference between HIT, HIT&P, and HVHIT (*p* ≥ 0.829) However, all the exercise groups differed significantly from the CG (*p* < 0.001), which deteriorated significantly (*p* = 0.039) during the study period (Figure 3).

Thus, hypothesis (a) that all the exercise programs were effective in improving the metabolic syndrome can be confirmed, but the result that all the protocols were similarly effective led to the rejection of hypothesis (b). Since hypothesis (c) was based on the theory that protein consumption increased the cardiometabolic effect of HIT, the corresponding issue was irrelevant.

### 3.2. Secondary Endpoints

Table 2 gives the results for secondary endpoints. Looking behind the covariates of the MetS (Table 2), MAP showed the most pronounced reductions (*p* ≤ 0.003) in the exercise groups. HDL-C (not given in Table 2) increased significantly (4.1 ± 5.6%, *p* = 0.001) in the HVHIT group only; however, calculating the more relevant total cholesterol/HDL-C ratio, all exercise groups improved significantly (*p* ≤ 0.003) and diverged (*p* ≤ 0.044) from the nontraining control group.

Due to the application of the percentage abdominal fat data that accounts for the varying weight changes of the groups, all exercise groups significantly lost abdominal fat (HIT: *p* < 0.001 to HIT&P: *p* = 0.029), although significant differences to control were only determined for the HIT group (*p* < 0.006).

In summary, no significant differences between HIT, HIT&P, and HVHIT were determined for any of the cardiometabolic parameters addressed.

### 3.3. Confounding/Explanatory Variables

Physical activity did not change relevantly, nor did exercise volume increase significantly in the groups (*p* > 0.171), at least after excluding participants who started such exercise outside the PUSH program (*n* = 5).

Despite the exclusion of 3 subjects with caloric reduction >10%, the energy consumption of the HIT group decreased significantly (*p* = 0.002) by 55 ± 101 kcal/d (−2.2 ± 4.1%), while the energy intake increased nonsignificantly by 0.2 ± 6.1 (HVHIT) to 1.7 ± 5.5% (CG). In parallel, dietary protein consumption decreased by −2.2 ± 11.4% (*p* = 0.192) in the HIT group and increased in the HIT&P (1.9 ± 13.3%; *p* = 0.574), HVHIT (1.6 ± 13.7%; *p* = 0.925), and the CG (3.7 ± 8.8%, *p* = 0.016). However, between-group differences were only observed for energy consumption (HIT versus CG: *p* = 0.027).

Potentially due to the latter factor, body mass changes at FU differed significantly (*p* = 0.001–0.005) between the HIT (−0.99 ± 2.35%, *p* = 0.009) and the HIT&P (1.52 ± 2.53%, *p* = 0.002) and HVHIT (1.47 ± 2.90%, *p* = 0.011) groups, while no relevant changes were observed in the CG (0.09 ± 2.57%, *p* = 0.975). LBM significantly increased in all exercise groups (HIT: 0.47 ± 1.32 kg; *p* = 0.035 versus HIT&P: 1.38 ± 1.45 kg; *p* = 0.001 versus HVT: 1.30 ± 1.42 kg; *p* = 0.001); however, significant differences (*p* = 0.001) to the nontraining CG (−0.04 ± 1.16 kg; *p* = 0.841) were determined for the HIT&P and HVT groups only.

## 4. Discussion

In general, the relevance of resistance exercise training for tackling cardiometabolic risk factors and diseases has been confirmed by several studies (review in [2, 3, 17–19]). However, much like pharmaceutical agents, exercise effects are dose dependent, with several parameters that can be varied. From a pragmatic point of view, besides exercise frequency, the duration of the exercise program may be an important criterion for people with low time budgets looking to improve their physical fitness, appearance, and/or general health.

In summary, whilst determining significant positive effects among the exercise groups, we did not observe significant differences between HIT, HIT&P, and HVHIT for the primary study endpoint MetS-*Z*-Score and related parameters. This finding was contrary to what we had expected, based on the superiority of multiple set exercise (HVT) in increasing lean body [8] and decreasing fat mass [20], and the corresponding positive association of both parameters with cardiometabolic risk including the MetS [21–24]. In fact, LBM changes were significantly lower in the HIT (0.56 ± 1.38 kg) compared with the HIT&P (1.38 ± 1.45 kg) and HVHIT group (1.42 ± 1.19 kg); however, abdominal body fat reductions were highest in the HIT group (Table 2). One may relate these results to the significant energy reduction (−55 ± 101 kcal/d) among the HIT group, but linear regression analysis determined only slight interactions between changes of energy intake and abdominal fat (adjusted *r* ^2^ = 0.11 and 0.03); also with respect to LBM we did not detect any corresponding interaction at all (adjusted *r* ^2^ = −0.007). Revisiting the beneficial effect of both parameters for cardiometabolic health, the harmful effect of abdominal, and specifically intra-abdominal, fat tissue is undisputed [22, 25, 26]. However, against the traditional belief of a generally beneficial effect of muscle tissue on cardiometabolic risk [21], recent findings by Kuk et al. [27] suggest “that, unlike visceral adipose tissue, whole-body skeletal muscle mass per se is not associated with either glucose tolerance or insulin sensitivity in overweight and obese men and women.” Although it is far beyond the scope of this paper to evaluate and discuss the pathways of resistance exercise induced cardiometabolic benefits in our cohort, linear regression adjusted for abdominal body fat changes determined a very low contribution of LBM increments on the MetS and related parameters (adjusted *r* ^2^ < 0.04). Correspondingly, group differences for LBM should not relevantly confound our result. In this context, additional protein supplementation that significantly increases HIT's effectiveness with respect to muscle gain did not result in more beneficial effects on any cardiometabolic parameter (Table 2) in the present cohort. This result was confirmed by 2 exercise studies [28, 29] that also focus on resistance exercise in (overweight) young to middle-aged males. Both studies evaluated the effect of additional supply of whey and/or soy protein (27 or 90 g/d) but did not observe significant improvements of the effect of their 6- or 12-week (HVT; ≈60–75% 1 RM) resistance exercise protocols on cardiometabolic risk factors related to the MetS. Unfortunately, the lack of resistance exercise trials with study arms comparable to the present study prevents a dedicated discussion of our HIT versus HVHIT issue.

In order to allow the reader to estimate the relevance, evidence, and generalizability of our results, some particular features and limitations of the study must be addressed. (1) We provided protein supplements in order to realize an overall protein intake of at least 1.5 g/kg body-mass/d, which is within the range (1.2–1.7 g/kg/d) suggested by the ACSM and ADA [30]. Although we are not aware of a direct independent effect of protein on cardiometabolic risk, the link between muscle mass and cardiometabolic risk suggests the relevance of adequate protein intake. However, the amount of protein intake to optimize muscle hypertrophy has yet to be determined [31]. Taking this uncertainty into account, we opted to increase the protein intake (1.0–1.1 g/kg/d) by ≈50% in order to generate a relevant difference in protein intake between the groups. (2) We applied a very sophisticated periodized resistance exercise protocol that may be somewhat exaggerated considering the untrained status of the participants. (3) We decided to use the METS-*Z*-Score on the basis of its higher sensitivity for relevant changes of modifiable risk factors compared with other indices (e.g., PROCAM [32]). (4) Two statistical limitations may reduce the evidence generated by the study. Most important, the slight but significant reduction of caloric uptake (55 ± 101 kcal/d) may have confounded our results, although linear regression determined only minor effects on our cardiometabolic outcomes at most. Further, the unexpectedly high number of CG participants who were unable or unwilling to conduct the subsequent HVHIT resistance exercise protocol decreases the statistical power of the study. (5) The 4-week delay between assessments and interventional start of the HVHIT group (Figure 2) was primarily based on our choice to avoid a second DXA assessment within 4 weeks and the Bavarian holidays, which prevented an earlier start of the intervention. We think this approach was reasonable at least with respect to radiation protection, although the dose applied by DXA is rather low for a whole body scan (<10 *µ*Sv; natural background exposure: ≈2400 *µ*Sv. p.a.).

However, we do not assume that this approach will have relevantly affected our results. (6) We focused on a homogeneous cohort of untrained and predominantly overweight middle-aged males in full-time employment for whom the relevance of this topic may be particularly high and of interest. Although we speculate that the results are transferable to other cohorts, with respect to the exhausting exercise protocol, female or elderly cohorts might not join corresponding HIT or HVHIT programs with much enthusiasm.

## 5. Conclusion

In summary, all present study protocols were similarly effective to improve cardiometabolic risk factors. However, taking the higher effect of HIT with additional protein supplementation on LBM into account, this strategy may be the best choice for people with low time budgets who want to improve their physical fitness, appearance, and/or general health.

## Figures and Tables

**Table 1:** Baseline characteristics of the PUSH study groups. No significant differences (*p* ≥ 0.30) between the groups were determined.

Variables	HIT group (*n* = 40)	HIT&P group (*n* = 40)	HVHIT group (*n* = 32)	Control (*n* = 40)
Age (y)	42.9 ± 5.4	43.7 ± 5.9	42.5 ± 5.6	42.5 ± 5.6
Body height (cm)	180.7 ± 7.2	180.9 ± 5.7	179.9 ± 7.4	180.4 ± 7.7
Body mass (kg)	90.1 ± 14.4	89.6 ± 14.6	87.1 ± 13.7	87.2 ± 15.7
Overweight (BMI > 25.0 kg/m^2^) (%)	67.5	70.0	57.5	55.0
Total body fat mass (%)^a^	25.6 ± 5.1	25.0 ± 4.8	24.9 ± 6.1	24.9 ± 6.0
Lean body mass (kg)^a^	67.1 ± 7.3	67.6 ± 8.4	64.0 ± 7.0	65.6 ± 7.8
Physical activity (index)^b^	2.9 ± 1.4	2.8 ± 1.3	2.7 ± 1.1	2.7 ± 1.1
Total exercise volume (min/week)	28.4 ± 35.9	40.1 ± 41.8	35.0 ± 37.9	32.1 ± 38.2
Energy intake (kcal/day)^c^	2658 ± 723	2703 ± 662	2588 ± 592	2516 ± 758
Fat/protein/carbohydrates (g/d)	99/90/305	106/94/314	100/92/286	94/89/298

^a^As assessed by Dual Energy X-Ray absorptiometry. ^b^Based on a scale from 1 (very low) to 7 (very high) according to a subjective assessment of professional, household, and recreational activities. ^c^Based on a 4-day dietary intake protocol.

**Table 2:** Effects of high intensity resistance exercise training (HIT) versus sedentary control (CG) on metabolic syndrome parameters. Intergroup differences were consistently adjusted for baseline values.

	HI(IT)	HIT & P	HVHIT	CG	Overall-*p* ^a^
Metabolic syndrome index (*Z*-Score)					
Baseline	−0.71 ± 2.94	−2.01 ± 2.42	−2.37 ± 3.21	−1.91 ± 3.07	0.101
Difference	−1.03 ± 1.56^*∗∗∗*^	−0.79 ± 0.91^*∗∗∗*^	−0.83 ± 0.76^*∗∗∗*^	0.31 ± 0.87^*∗*^	<0.001

Waist circumference (cm)					
Baseline	101.4 ± 10.6	99.7 ± 11.3	95.5 ± 11.3	97.1 ± 11.0	0.151
Difference	−1.60 ± 2.36^*∗∗∗*^	−0.95 ± 2.37^*∗*^	−0.92 ± 2.40	0.09 ± 1.88^n.s^	0.018
Mean arterial pressure (MAP) (mm/Hg)					
Baseline	100.7 ± 10.1	108 ± 8.5	97.4 ± 8.3	99.0 ± 9.5	0.406
Difference	−3.84 ± 3.90^*∗∗∗*^	−2.27 ± 3.68^*∗∗∗*^	−2.22 ± 3.55^*∗∗*^	0.09 ± 2.87^n.s^	<0.001
Triglycerides (mg/dL)					
Baseline	168.3 ± 77.6	150.1 ± 70.2	161.5 ± 80.1	146.0 ± 74.9	0.582
Difference	−5.5 ± 35.4^n.s.^	−22.0 ± 41.9^*∗∗*^	−12.5 ± 30.6^*∗*^	23.9 ± 39.6^*∗∗∗*^	<0.001
Fasting glucose (mg/dL)					
Baseline	97.8 ± 18.5	89.1 ± 7.7	92.4 ± 13.9	95.4 ± 14.3	0.582
Difference	−2.41 ± 6.33^*∗*^	−1.65 ± 5.63^n.s.^	−2.07 ± 4.16^*∗*^	−0.19 ± 5.27^n.s^	0.349
Cholesterol/high density lipoprotein Cholesterol (mg/dL)					
Baseline	4.81 ± 1.17	4.25 ± 0.82	4.26 ± 1.12	4.65 ± 1.28	0.091
Difference	−0.23 ± 0.42^*∗∗*^	−0.20 ± 0.31^*∗∗*^	−0.21 ± 0.26^*∗∗∗*^	0.03 ± 0.34^n.s^	0.005
Abdominal body fat (%)					
Baseline	29.8 ± 6.9	28.3 ± 7.6	26.9 ± 9.4	27.9 ± 8.9	0.425
Difference	−1.42 ± 2.09^*∗∗∗*^	−0.86 ± 2.32^*∗*^	−0.83 ± 1.72^*∗*^	0.38 ± 2.01^n.s^	0.003

Asterisk (*∗*) indicates changes within the group: ^*∗*^ *p* < 0.05; ^*∗∗*^ *p* < 0.01; ^*∗∗∗*^ *p* < 0.001; n.s.: nonsignificant. ^a^Results of post hoc tests with dedicated comparisons between the groups are listed in the text.
